# Buccal Bone Wall Thickness Dictates the Extent of Vertical Buccal Bone Loss Following Implant Placement: A Preclinical Study

**DOI:** 10.1111/jcpe.70027

**Published:** 2025-08-29

**Authors:** Jean‐Claude Imber, Andrea Roccuzzo, Alberto Monje, Benjamin Pippenger, Dieter D. Bosshardt, Anton Sculean, Daniel Buser

**Affiliations:** ^1^ Department of Periodontology, School of Dental Medicine University of Bern Bern Switzerland; ^2^ Shanghai Perio‐Implant Innovation Center, Institute of Integrated Oral, Craniofacial and Sensory Research, Shanghai Ninth People's Hospital, Shanghai Jiao Tong University School of Medicine Shanghai China; ^3^ College of Stomatology, Shanghai Jiao Tong University, National Center of Stomatology, National Clinical Research Center for Oral Diseases, Shanghai Key Laboratory of Stomatology Shanghai China; ^4^ Department of Periodontology and Oral Medicine University of Michigan Ann Arbor Michigan USA; ^5^ Department of Periodontology Universitat Internacional de Catalunya Barcelona Spain; ^6^ Department of Surgical Research AnaPath Services Liestal Switzerland; ^7^ School of Dental Medicine, University of Bern Bern Switzerland; ^8^ Centre for Implantology Buser and Frei Bern Switzerland

**Keywords:** animal model, bone resorption, buccal bone wall, dental implants, histology

## Abstract

**Aim:**

To assess buccal vertical bone resorption following implant placement in healed sites with varying buccal bone wall thicknesses.

**Materials and Methods:**

In 11 miniature pigs, three tapered hybrid titanium implants were placed per hemi‐maxilla in healed bone. Sites were randomised into three groups based on buccal bone wall thickness: G1 (< 1.0 mm), G2 (1.0–1.5 mm) and G3 (> 1.5 mm). Animals were euthanised at 24 h and 2, 4 or 8 weeks. Histological and histometric analyses were performed. The primary outcome was the vertical distance from the transition point (TP) between the machined and moderately rough implant surfaces to the first bone‐to‐implant contact (fBIC).

**Results:**

Healing was uneventful. At 2 weeks, all groups showed buccal resorption, although G3 exhibited earlier bone apposition and fewer resorptive signs. By 8 weeks, all G1 implants displayed exposure of the moderately rough surface, while only one implant in G3 showed exposure. TP‐fBIC values at 8 weeks were 0.92 ± 0.63 mm (G1), 0.27 ± 0.54 mm (G2, *p* = 0.041) and −0.16 ± 0.17 mm (G3, *p* = 0.0002). Bone‐to‐implant contact increased over time across all groups.

**Conclusion:**

Thin buccal bone walls (< 1 mm) were associated with greater vertical bone loss and implant surface exposure, whereas thick walls (> 1.5 mm) preserved the buccal bone better and protected the implant surface.

## 1 Introduction

Nowadays, dental implants are a reliable treatment option with predictable long‐term results in partially and fully edentulous patients (Buser et al. 2012; Buser, Sennerby, and De Bruyn 2017; Ducommun et al. 2019; Duong et al. 2022; Roccuzzo, Imber, Marruganti, et al. 2022). Implant placement is performed either after extraction or in healed sites, and clinicians have many treatment options from a surgical standpoint (Buser, Chappuis, et al. 2017). Currently, a significant proportion of dental implants are placed in partially or fully healed post‐extraction sites—following a period of soft‐ and/or hard‐tissue healing—rather than in fresh extraction sockets (Buser, Sennerby, and De Bruyn 2017). Significant progress has been achieved through preclinical and clinical studies that have extensively investigated alveolar ridge alterations following tooth extraction. Araújo and Lindhe (2005) have systematically evaluated and described the sequential phases of soft‐tissue healing and bundle bone resorption following tooth extraction in mandibular premolar sites of beagle dogs, using histological sections obtained at 2‐week intervals, providing a thorough understanding of tissue biology in post‐extraction healing.

Postoperative bone resorption and bone remodelling following implant placement in healed sites is a well‐documented but not fully understood process. In addition to the surgical trauma, the local bone anatomy plays an important role in the amount of bone resorption, including the shape of the alveolar ridge, structure of the bone and, most importantly, the thickness of the oral and buccal bone walls after implant site preparation (Araújo et al. 2023; Barone et al. 2016; Covani et al. 2004; Liñares et al. 2023; Mardas, Macbeth, et al. 2023; Monje et al. 2019; Spray et al. 2000; Suaid et al. 2014). Clinical and preclinical observations have shown that a thin buccal bone wall is susceptible to postoperative resorption, leading to an exposure of the rough implant surface, often combined with subsequent recession of the peri‐implant mucosa (Jensen et al. 2023; Monje et al. 2019, 2023). In addition, early bone loss post implant placement—leading to an exposed rough implant surface—has been recently identified by two clinical studies as a predictor for the development of peri‐implantitis (Ravidà et al. 2023; Windael et al. 2021). Both complications have negative consequences for the patient, require additional therapies and can lead to reduced survival and success rates. Hence, early bone loss should be prevented.

The extent to which early bone loss following implant surgery is influenced by the thickness of the buccal bone wall remains incompletely understood, despite its high clinical relevance in daily practice. Therefore, the primary objective of this preclinical study was to evaluate the extent of early vertical bone resorption during a 2‐month healing period following implant placement in healed sites with varying buccal bone wall thicknesses. The secondary objective of the study was to evaluate the dynamics and pattern of this post‐surgical bone resorption at different time points during an 8‐week period.

## 2 Materials and Methods

### 2.1 Study Design and Sample Size

Eleven female miniature pigs (weight: approx. 40 kg; age: 22–26 months at tooth extraction) with intact dentition and healthy periodontal status were used. Housed under laboratory conditions (15°C–21°C, > 30% humidity), they had ad libitum access to tap water and a soft laboratory diet. The study was approved by the Danish Ministry of Food, Agriculture and Fisheries (approval no. 2021‐15‐0201‐00876), complied with the Danish Animal Protection Law and ARRIVE guidelines (du Sert et al. 2020) and adhered to the 3Rs (Replace, Reduce, Refine) principles.

Sample size was determined by a power calculation based on vertical bone resorption data from a previous study (Monje et al. 2019).

**FIGURE 1:**
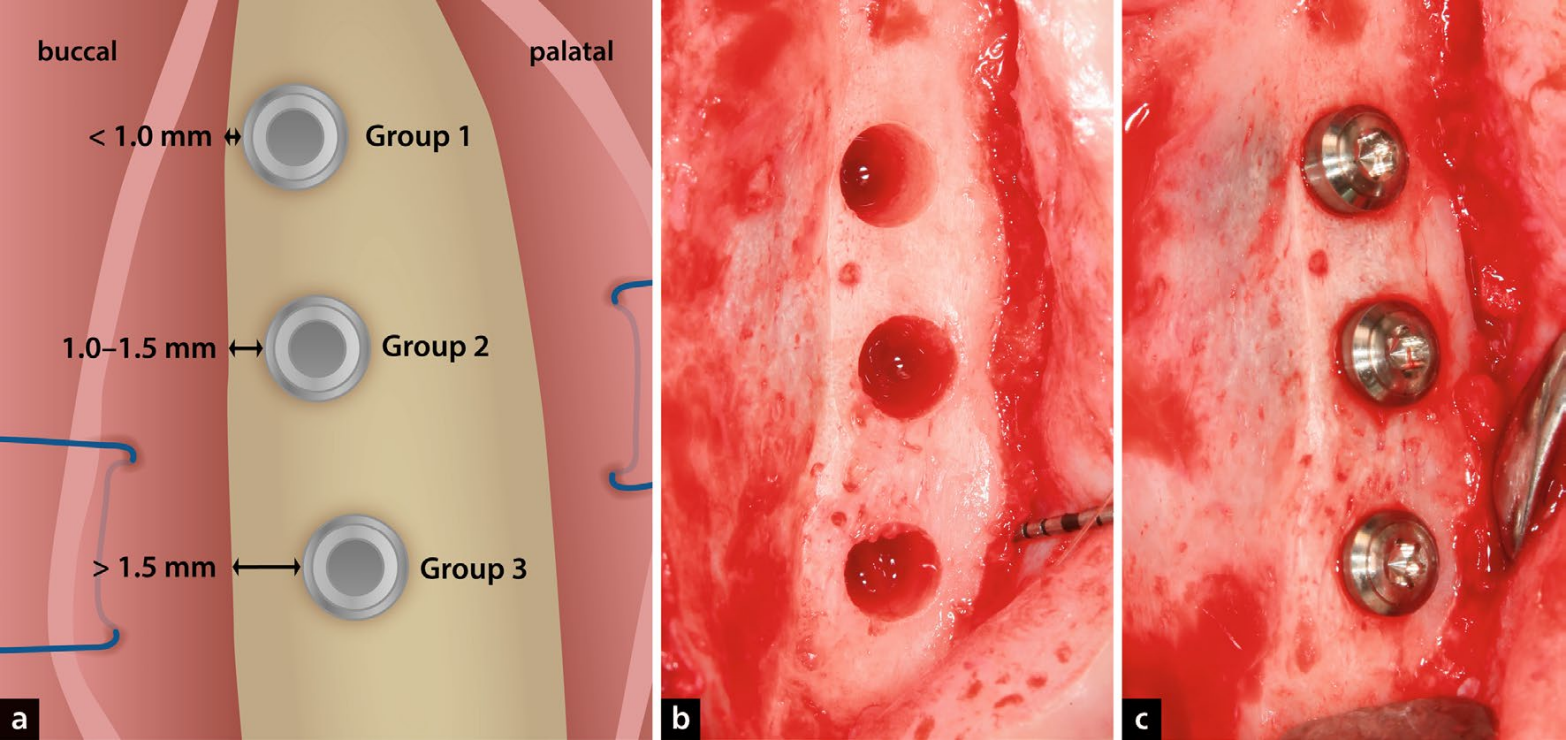
Graphical illustration (a) and clinical pictures (b, c) illustrating the procedure.

The study followed a randomised design with three experimental groups per hemi‐maxilla and four healing periods. Group allocation was randomised to ensure balanced distribution across groups and time points. Implant positions (mesial, central, distal) were evenly assigned within each hemi‐maxilla to minimise positional bias (Figure 1).

Group 1 (G1): Thin buccal bone wall thickness (< 1.0 mm)

Group 2 (G2): Medium buccal bone wall thickness (1.0–1.5 mm)

Group 3 (G3): Thick buccal bone wall thickness (> 1.5 mm)

**FIGURE 2:**
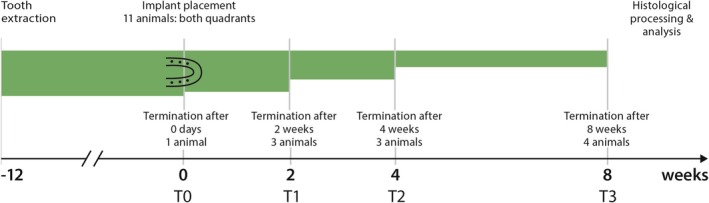
Timetable.

Healing times were 0 days (T0, one animal), 2 weeks (T1, two animals), 4 weeks (T2, three animals) and 8 weeks (T3, four animals). One animal was allocated to T0 to confirm standardised implant placement and buccal bone wall thickness immediately after surgery. This served as a baseline reference to ensure that the observed changes at later time points reflected biological remodelling rather than surgical variability, in accordance with the 3R principle of reduction. The study timeline is shown in Figure 2. To minimise bias, animal caretakers, the supervising veterinarian and the histologists (J.‐C.I. and D.D.B.) were blinded to group assignments.

### 2.2 Surgical Procedure

All surgeries were performed under general anaesthesia and aseptic conditions by an experienced surgeon (J.‐C.I.). Animals were fasted overnight and pre‐anesthetised. After general anaesthesia, local anaesthesia with lidocaine/adrenaline (Xylocain Dental Adrenalin 20 mg/mL + 12.5 mg/mL) reduced perioperative discomfort and bleeding. Post operation, the animals received Vetergesic (0.3 mg/mL, at a dose of 0.1 mL/kg, IV/IM). All animals received an oral suspension of Streptocillin (0.1 mL/kg, IM) until Day 7 post surgery and Metacam (15 mg/mL, at a dose of 0.03 mL/kg) until Day 5 post surgery. All procedures were supervised by veterinarians.

In the first surgical phase, all maxillary incisors (three incisors per hemi‐maxilla; six incisors per animal) were extracted without flap elevation and allowed to heal for 12 weeks. In the second phase, mucoperiosteal flaps were elevated bilaterally, and the alveolar ridge was flattened on the occlusal aspect with rotary instrumentation under copious irrigation to standardise the buccal bone wall (Figure 1b). Six dental implants were placed per animal using a surgical stent. Buccal wall thickness was verified with a calliper. The implants (Straumann TLC, Ø3.3 mm, length 8 mm, Roxolid, SLActive, SP/NT) had a hybrid surface: a moderately rough, hydrophilic endosseous portion and a machined trans‐crestal collar. The SLActive surface was placed fully in bone, and the 1.8‐mm machined collar was positioned ~1 mm subcrestally, leaving the implant shoulder ~0.8 mm supracrestally (Figure 1c). Closure screws were inserted, and flaps were repositioned for submerged healing. Animals were euthanised at allocated time points via intracardiac injection of pentobarbital.

### 2.3 Histological Procedures

After euthanasia, maxillae were harvested and tissue blocks fixed in formaldehyde. All 22 hemi‐maxillae were dehydrated in graded ethanol, then embedded in methyl methacrylate (MMA). After polymerisation, specimens were sectioned oro‐facially along the implant axis using a slow‐speed diamond saw with coolant (Varicut VC‐50; Leco, Germany). Three ground sections per implant were prepared; two ~800‐μm sections were mounted on Plexiglas and ground to 150 μm (Knuth‐Rotor‐3; Struers, Denmark). Sections were stained with toluidine blue/McNeal and basic fuchsin. High‐resolution images were obtained using a light microscope (Axio Imager M2; Zeiss) and Zen 2.6 Pro software; key areas were additionally imaged with a digital camera (Gryphax, Jenoptik) mounted on the microscope.

### 2.4 Histometric Analysis

The central sections showing the widest implant diameter were selected for histometric analysis, following the protocol of Janner et al. (2018). The following histometric landmarks (Figure S1) were identified on both buccal and palatal sides by two investigators (D.D.B. and J.‐C.I.). Discrepancies were resolved by discussion.

– Outer implant surface (S)
– Implant shoulder (IS)
– Transition point between the machined and moderately rough implant surfaces (TP)
– Start of the apical implant curvature (SAC)
– Outer contour of the buccal bone wall (OCB)
– First bone‐to‐implant contact (fBIC)

As the TP was not always clearly visible, it was defined as 1.8 mm apical to the implant shoulder (IS), corresponding to the machined collar height. The following vertical and horizontal histometric measurements were performed on the buccal and palatal aspects using the Zeiss Zen 2.6 Pro software.

– TP–fBIC: vertical distance between TP and the fBIC in mm
– S–OCB: horizontal distance from S to the OCB (in mm) at 4 different levels: at the level of the TP and then 1, 2 and 3 mm apical to TP

BIC was assessed from TP to the start of the apical implant curvature (SAC) on both buccal and palatal sides, as well as total BIC (buccal + palatal). The following parameters were quantified as percentages of the implant surface: soft tissues, coagulum, bone debris, osteoid, new bone and old bone. Total BIC was defined as the sum of osteoid, new bone and old bone.

### 2.5 Statistical Analysis

Statistical analyses were conducted in R (v4.3.2). Continuous length outcomes were summarised by means, SDs, minima and maxima, and nominal BIC outcomes were reported as percentages. Length data were analysed at the implant level using linear mixed models with fixed effects for time (2w, 4w, 8w), group and their interaction, and specimen as a random factor (random intercept ± slopes by group). Model fit was assessed via residual and random effect normality (Shapiro–Wilk test) and DHARMa goodness‐of‐fit tests (Hartig 2018). Fixed effects were tested with Type III ANOVA using Satterthwaite's approximation; significant effects underwent post hoc Satterthwaite *t*‐tests with Holm correction. BIC outcomes were analysed with mixed logistic regression models including the same fixed effects and a random intercept for specimen. For combined data, Side (buccal, palatal) was added as a fixed factor. Overdispersion was addressed with an observation‐level random effect. Model fit was evaluated via Pearson residual dispersion and DHARMa tests. Fixed effects were tested using Wald Type III ANOVAs; significant effects were followed by post hoc Wald *z*‐tests with Holm correction. A *p*‐value ≤ 0.05 was considered significant.

## 3 Results

### 3.1 Clinical Findings

Healing was uneventful, with no infections or complications observed. All implants remained submerged until euthanasia. A total of 66 implants (6 per animal, 11 animals) were placed, yielding 22 implants per group for histological analysis.

### 3.2 Descriptive Histology

**FIGURE 3:**
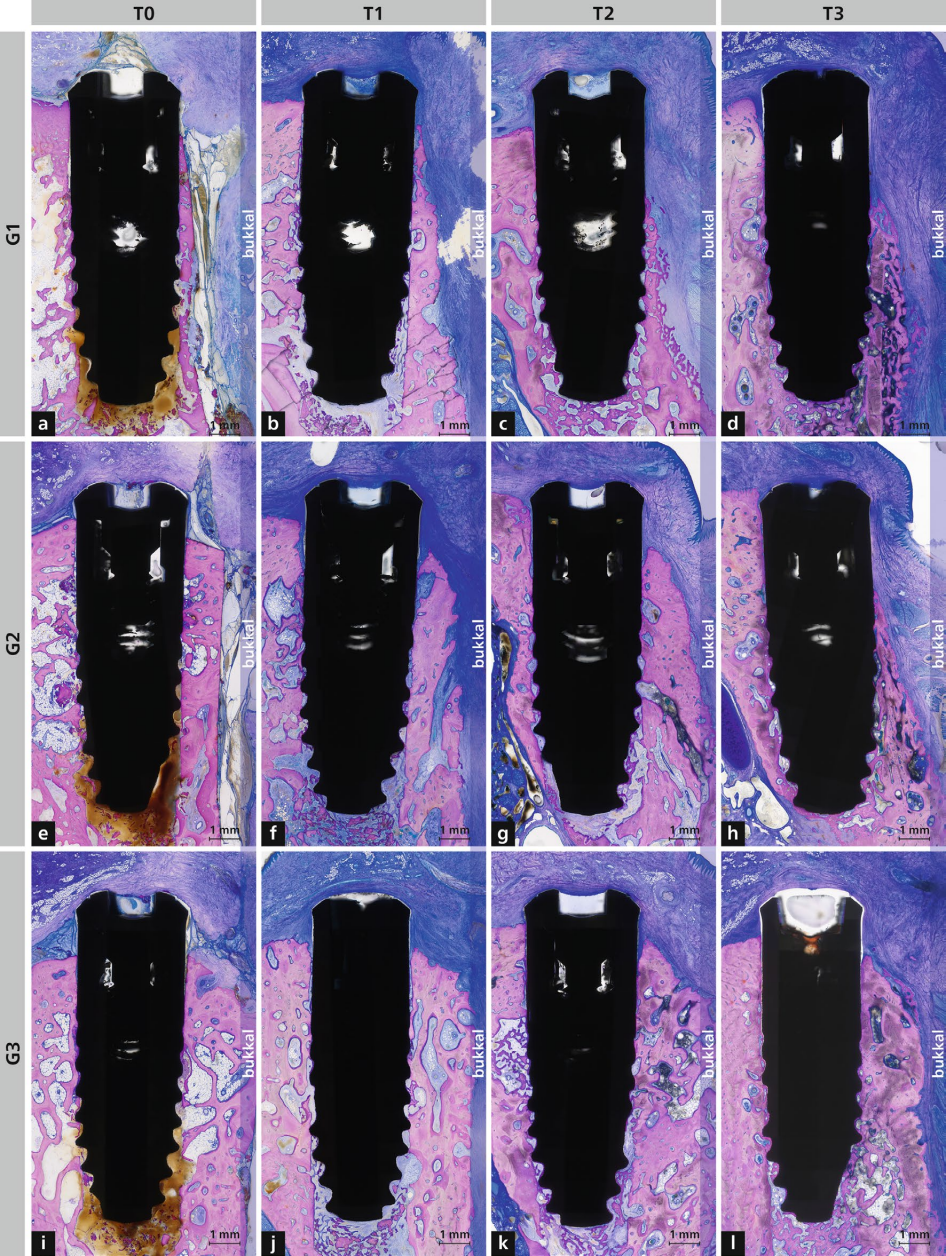
Representative histological sections of Group 1 (G1; a–d), Group 2 (G2; e–h) and Group 3 (G3; i–l) at four healing time points: T0 (0 days), T1 (2 weeks), T2 (4 weeks) and T3 (8 weeks). Staining: Toluidine blue/McNeal + basic fuchsin.

A total of 198 ground sections (three per implant) were prepared, of which 122 were mounted and processed on Plexiglas. Representative sections are shown in Figure 3 and Figures [Link], [Link]. Processing artefacts were rare and did not affect the evaluations.

At T0, all six implants showed contact to existing bone, standardised buccal bone wall thickness (Figure 3a,e,i and Figure S2) and consistent insertion depth. Bone debris and coagulum were present between threads and apically. Of the 60 implants placed at T1 to T3, 58 showed ongoing osseointegration. One implant (G3 at T3) perforated the nasal floor and was associated with local inflammation; another (also G3 at T3) was largely in the nasal floor with minimal bone contact. At T1 to T3, the coronal non‐intraosseous parts were consistently covered by soft connective tissue, with intact epithelium and no dehiscence (Figure 3). Seven implants (G1: T1, T3; G2: T1, T3; G3: T1, T2, T3) were partially located in the palatal nasal floor, reducing the osseointegrated surface. Four implants (G2: T1, T2; G3: T1, T2) had reduced palatal osseointegration due to placement in a growth suture, allowing soft‐tissue ingrowth.

**FIGURE 4:**
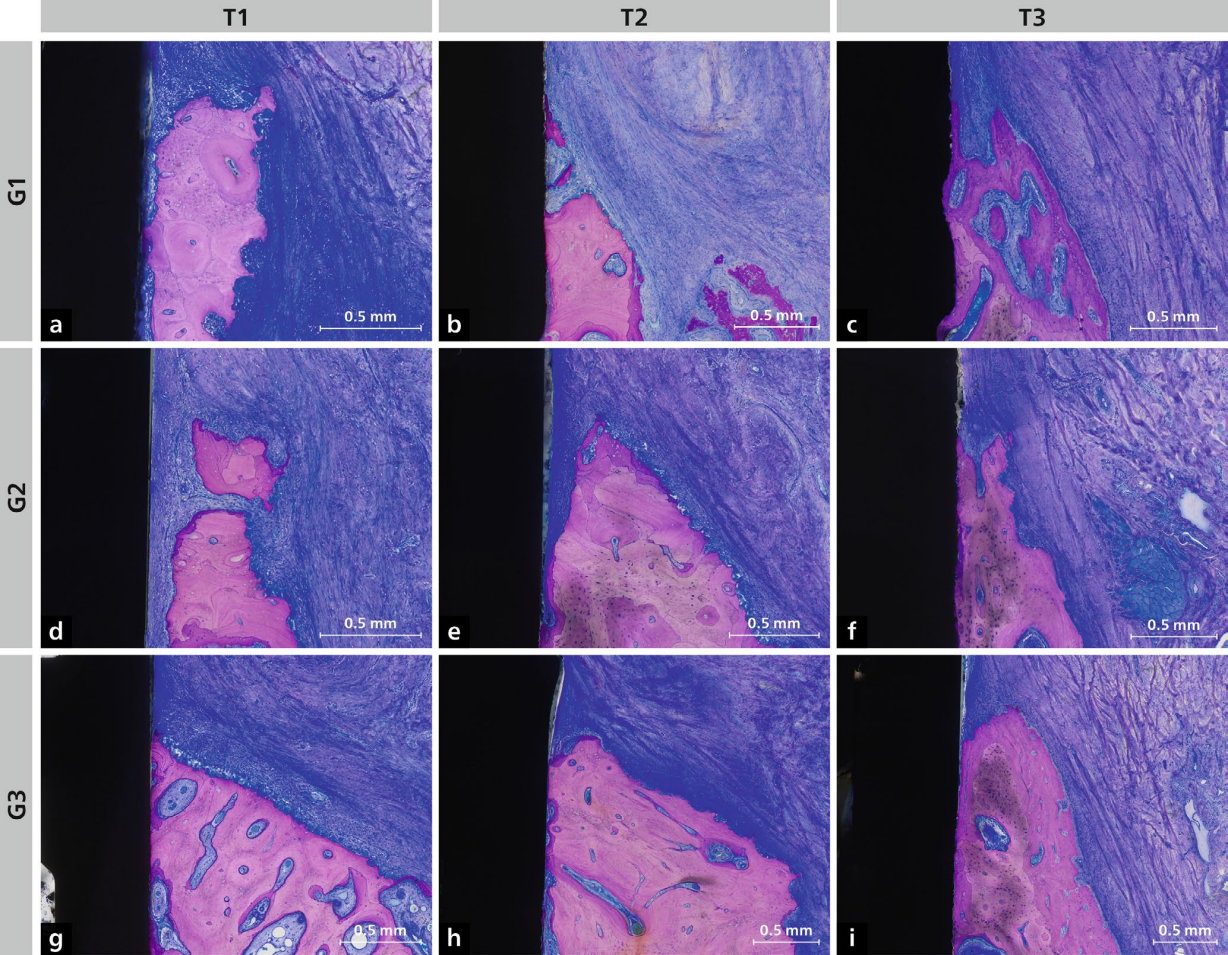
High‐magnification views of the buccal bone crest in Group 1 (G1; a–c), Group 2 (G2; d–f) and Group 3 (G3; g–i) at T1 (2 weeks), T2 (4 weeks) and T3 (8 weeks), respectively. Staining: toluidine blue/McNeal + basic fuchsin.

At T1 (Figure 3b,f,j and Figure S3), the outer surface of the bone adjacent to the implant had not yet re‐established a periosteum, and many ongoing resorption processes with numerous osteoclasts were visible in all specimens (Figure 4a,d,g). Some small areas of the outer surface of the bone showed signs of bone formation with osteoblasts. In the intraosseous part of the implants, progressive osseointegration with the formation of woven bone was observed.

At T2 (Figure 3c,g,k and Figure S4), sites with resorption processes were reduced and there were regions with bone apposition with osteoblasts and sites with bone resorption and osteoclasts (Figure 4b,e,h). On the palatal side of the implants, the bone level was comparable between the three groups. In contrast, on the buccal side, G1 showed more advanced vertical bone loss and therefore more exposed implant surface to soft connective tissue. Osseointegration was more established at T2 compared to T1, and some areas were already covered by more mature new bone.

At T3 (Figure 3d,h,l and Figure S5), resorption was nearly absent, and a periosteum had re‐formed on parts of the outer bone wall. Bone‐forming cells outnumbered osteoclasts (Figure 4c,f,i). In G1, all implants showed varying degrees of exposure of the moderately rough surface. In G2, only one implant had complete bone coverage of the rough surface, while in G3, only one implant showed surface exposure.

### 3.3 Histometric Analysis

One implant (G3 at T3) was excluded from histometric analysis because of infection, and another (G3 at T3) was excluded because of severe nasal floor perforation. For four implants (G2 at T1 and T2, G3 at T1 and T2), the palatal measurement from TP to fBIC was not possible because of a growth line leading to soft‐tissue ingrowth in this region. Consequently, the area for BIC evaluation was reduced for these four implants. Seven implants (G1 at T1 and T3; G2 at T1 and T3; G3 at T1, T2, and T3) were partially in the nasal floor on the palatal side, which reduced the area to evaluate BIC.

### 3.4 Vertical Measurements

Vertical measurements are summarised in Table 1. For the primary outcome (buccal TP–fBIC), significant effects were found for time (*p* = 0.002) and group (*p* < 0.0001), with no interaction (*p* = 0.18). G1 showed a significant change from 2 to 8 weeks (−0.952 mm, *p* = 0.0026). Significant differences were also found between G1 and G2 and between G1 and G3 at T2 and T3, with the largest effect between G1 and G3 at T3 (1.077 mm, *p* = 0.0002). No significant effects were observed for palatal TP–fBIC (time: *p* = 0.16; group: *p* = 0.34; interaction: *p* = 0.60), so no post hoc tests were conducted.

**TABLE 1:** Histometry—Vertical measurements.

	T1 (2 weeks)		T2 (4 weeks)		T3 (8 weeks)	
	TP‐fBIC buccal (mm), mean ± SD	TP‐fBIC palatal (mm), mean ± SD	TP‐fBIC buccal (mm), mean ± SD	TP‐fBIC palatal (mm), mean ± SD	TP‐fBIC buccal (mm), mean ± SD	TP‐fBIC palatal (mm), mean ± SD
	Min.	Min.	Min.	Min.	Min.	Min.
	Max.	Max.	Max.	Max.	Max.	Max.
	*n*	*n*	*n*	*n*	*n*	*n*
G1	0.03 ± 0.2 –0.16 0.33 6	0.23 ± 1.08 –1.32 1.57 6	–0.52 ± 0.42 –1.28 –0.1 6	0.23 ± 0.63 –0.67 1.15 6	–0.92 ± 0.63 –1.62 –0.14 8	0.35 ± 1.13 –1.75 1.71 8
G2	0.06 ± 0.58 –0.3 0.93 6	0.13 ± 0.73 –0.88 0.84 5	0.23 ± 0.41 –0.14 0.93 6	0.83 ± 0.5 0.1 1.43 5	–0.27 ± 0.54 –0.97 0.74 8	1.04 ± 0.66 –0.32 1.67 8
G3	0.38 ± 0.32 0.04 0.87 6	–0.04 ± 0.54 –0.99 0.35 5	0.35 ± 0.43 –0.23 1.05 6	0.86 ± 0.6 –0.04 1.4 5	0.16 ± 0.17 –0.11 0.48 6	0.73 ± 0.65 –0.13 1.61 6
*p*‐value G1 versus G2	1	n.s.	0.041	n.s.	0.041	n.s.
*p*‐value G1 versus G3	0.782	n.s.	0.009	n.s.	0.0002	n.s.
*p*‐value G2 versus G3	0.847	n.s.	1	n.s.	0.283	n.s.

Abbreviations: fBIC, first bone‐to‐implant contact; G1, thin buccal bone wall thickness (< 1.0 mm); G2, medium buccal bone wall thickness (1.0–1.5 mm); G3, thick buccal bone wall thickness (> 1.5 mm); *n*, number of implants; n.s., not significant; SD, standard deviation; TP, transition point between the machined and moderately rough implant surfaces.

### 3.5 Horizontal Measurements

Horizontal measurements are summarised in Table 2. For bone thickness at TP (S‐OCB), values were highest in G3, followed by G2, and lowest in G1 across all time points. A significant group effect was found (*p* = 0.007), but no effect of time (*p* = 0.95) or interaction (*p* = 0.93); post hoc comparisons were not significant (*p* ≥ 0.12).

**TABLE 2:** Histometry—Horizontal measurements.

Timepoint	Group	S‐OCB at TP (mm), mean ± SD	S‐OCB at TP –1 mm (mm), mean ± SD	S‐OCB at TP –2 mm (mm), mean ± SD	S‐OCB at TP –3 mm (mm), mean ± SD
		Min.	Min.	Min.	Min.
		Max.	Max.	Max.	Max.
T1 (2 weeks)	G1 (*n* = 6)	0.34 ± 0.13 0.13 0.52	0.37 ± 0.15 0.15 0.53	0.54 ± 0.19 0.24 0.73	0.72 ± 0.35 0.26 1.28
	G2 (*n* = 6)	0.66 ± 0.3 0.45 1.23	1.11 ± 0.42 0.31 1.5	1.42 ± 0.24 1.14 1.72	1.79 ± 0.45 1.21 2.25
	G3 (*n* = 6)	1.15 ± 0.4 0.57 1.6	1.91 ± 0.49 1.29 2.75	2.0 ± 0.4 1.36 2.5	1.93 ± 0.56 1.23 2.81
	*p*‐value G1 versus G2	0.844	0.168	0.1283	0.134
	*p*‐value G1 versus G3	0.489	0.005	0.002	0.007
	*p*‐value G2 versus G3	0.898	0.365	0.396	0.718
T2 (4 weeks)	G1 (*n* = 6)	0.32 ± 0.26 0 0.57	0.67 ± 0.64 0 1.62	0.89 ± 0.62 0.16 1.68	1.1 ± 0.82 0.12 2.22
	G2 (*n* = 6)	0.6 ± 0.35 0.24 1.23	1.21 ± 0.48 0.55 2.01	1.66 ± 0.37 1.01 2.03	1.9 ± 0.4 1.45 2.37
	G3 (*n* = 6)	1.01 ± 0.48 0.52 1.91	1.76 ± 0.52 1.03 2.34	2.23 ± 0.6 1.54 3.16	2.68 ± 0.71 2 3.72
	*p*‐value G1 versus G2	0.898	0.365	0.15	0.253
	*p*‐value G1 versus G3	0.672	0.038	0.003	< 0.0001
	*p*‐value G2 versus G3	0.898	0.458	0.396	0.253
T3 (8 weeks)	G1 (*n* = 8)	0.19 ± 0.28 0 0.63	0.59 ± 0.46 0 1.27	0.94 ± 0.32 0.5 1.31	1.25 ± 0.26 0.89 1.65
	G2 (*n* = 8)	0.45 ± 0.34 0 0.92	0.93 ± 0.6 0 1.72	1.39 ± 0.63 0.34 2.44	1.61 ± 0.62 0.51 2.33
	G3 (*n* = 6)	1.29 ± 0.97 0.25 2.97	1.87 ± 0.72 0.85 2.91	2.21 ± 0.62 1.45 2.97	2.35 ± 0.72 1.41 3.22
	*p*‐value G1 versus G2	0.898	0.458	0.396	0.718
	*p*‐value G1 versus G3	0.12	0.006	0.002	0.007
	*p*‐value G2 versus G3	0.44	0.1681	0.132	0.235

Abbreviations: G1, thin buccal bone wall thickness (< 1.0 mm); G2, medium buccal bone wall thickness (1.0–1.5 mm); G3, thick buccal bone wall thickness (> 1.5 mm); *n*, number of implants; OCB, outer contour of the buccal bone wall; S, outer implant surface; SD, standard deviation; TP, transition point between the machined and moderately rough implant surfaces.

At 1 mm apical to TP, group differences were highly significant (*p* < 0.0001), with no time (*p* = 0.93) or interaction effects (*p* = 0.70). All G1 versus G3 comparisons were significant (*p* < 0.04), with differences ≥ 1.089 mm.

At 2 mm apical, group effects remained significant (*p* < 0.0001), with no time (*p* = 0.47) or interaction effects (*p* = 0.81). All G1 versus G3 comparisons were significant (*p* < 0.003), with differences ≥ 1.241 mm.

At 3 mm apical, a significant group effect was again observed (*p* < 0.0001), without time (*p* = 0.38) or interaction effects (*p* = 0.45). G1 versus G3 comparisons were all significant (*p* < 0.0074), with differences ≥ 1.055 mm.

### 3.6 Bone‐To‐Implant Contact

Values of the total BIC (osteoid + new bone + old bone) are shown in Table 3. All BIC scores are summarised in Table S1. The total BIC values increased over time in all groups but only reached statistical significance in G2 from T1 to T2 (76.7%–84.7%, *p* = 0.027) and in G3 from T1 to T3 (78.5%–88%, *p* = 0.003) and from T2 to T3 (82.2%–88%, *p* = 0.037). When comparing the groups during the different time points, only G1 compared to G3 at T3 reached significance (80.2% vs. 88%, *p* = 0.002).

**TABLE 3:** Total BIC analysis.

	Total BIC, % (osteoid + new bone + old bone)			*p*		
	T1 (2 weeks)	T2 (4 weeks)	T3 (8 weeks)	T1 versus T2	T1 versus T3	T2 versus T3
Group						
G1	73.2 (*n* = 6)	81.7 (*n* = 6)	80.2 (*n* = 8)	0.118	0.182	0.992
G2	76.7 (*n* = 6)	84.7 (*n* = 6)	83.9 (*n* = 8)	0.027*	0.145	0.992
G3	78.5 (*n* = 6)	81.2 (*n* = 6)	88.0 (*n* = 6)	0.992	0.003*	0.037*
*p*‐value						
G1 versus G2	1	0.582	0.733			
G1 versus G3	0.583	1	0.002*			
G2 versus G3	1	0.458	0.121			

Abbreviations: BIC, bone‐to‐implant contact; G1, thin buccal bone wall thickness (< 1.0 mm); G2, medium buccal bone wall thickness (1.0–1.5 mm); G3, thick buccal bone wall thickness (> 1.5 mm); *n*, number of implants; OCB, outer contour of the buccal bone wall; S, outer implant surface; SD, standard deviation; TP, transition point between the machined and moderately rough implant surface.

* *p* < 0.05.

## 4 Discussion

This preclinical study evaluated buccal bone dimensional changes over 8 weeks following implant placement in healed sites with varying buccal bone thicknesses. Histology showed group‐specific resorption patterns: at 2 weeks, all groups displayed osteoclastic activity without differences in vertical bone loss. By 8 weeks, Group 1 (< 1.0 mm) exhibited significant vertical resorption and implant surface exposure; Group 2 (1.0–1.5 mm) had moderate resorption with less frequent, less pronounced exposure; and Group 3 (> 1.5 mm) showed minimal resorption in eight of nine implants with no exposure. Linear fBIC–TP measurements at 8 weeks were 0.92 ± 0.63 mm (Group 1), 0.27 ± 0.54 mm (Group 2) and −0.16 ± 0.17 mm (Group 3). Since the TP was placed ~1 mm subcrestally, Group 1 showed nearly 2.0 mm of vertical bone loss. Given that TP was placed ~1 mm subcrestally, Group 1 showed an adjusted mean vertical bone loss of nearly 2.0 mm at 8 weeks.

These findings are consistent with those of Monje et al. (2019), who identified 1.5 mm as the critical buccal bone thickness for crest stability. After 8 weeks, implants in thin walls (< 1.5 mm) showed 3.7 mm of vertical bone loss, while no loss occurred in the thick‐wall group (≥ 1.5 mm; *p* < 0.001). The greater loss in Monje's study may reflect differences in implant design, animal model (dog vs. miniature pig) or site (posterior mandible vs. anterior maxilla). Unlike that study, the present one offers detailed insight into bone resorption and healing dynamics over three time points, supported by histology and histometric measurements.

Vertical bone loss in thin buccal walls may be explained by the ‘avascular necrosis’ theory (Mankin 1992; Monje et al. 2019), where flap elevation and implant insertion compromise both periosteal and endosteal blood supply, leading to cortical bone necrosis and resorption. Osteoclast‐mediated resorption, likely via the RANKL/RANK pathway and NFAT signalling (Roux and Orcel 2000), is implicated. Our histology at 2 and 4 weeks confirmed marked osteoclastic activity in Groups 1 and 2, supporting this mechanism.

At 8 weeks, all implants in Group 1 and most in Group 2 showed exposure of the moderately rough implant surface, while only one implant in Group 3 showed minimal exposure. This finding has important clinical implications, as exposure of the rough surface to the peri‐implant sulcus facilitates bacterial colonisation and may trigger peri‐implant diseases such as mucositis and peri‐implantitis (El Kholy et al. 2020). Peri‐implantitis is a prevalent, site‐specific complication in implant dentistry (Berglundh et al. 2024; Rakic et al. 2018; Roccuzzo et al. 2023), and local factors such as surface exposure play a critical role in its onset and progression.

These preclinical findings support the design rationale of hybrid implants, which feature a moderately rough endosseous surface and a smooth, machined transcrestal collar (Buser 2022; Tarnow 1993). In this model, hybrid implants showed high bone‐to‐implant contact, and in sites with thicker buccal bone, the rough surface remained fully covered throughout the study. Although not designed to assess implant performance or clinical outcomes, the maintained hard‐tissue coverage aligns with the collar's intended function. While clinical studies report varying complication rates for different implant designs (Buser et al. 2012; Derks et al. 2016; Windael et al. 2021), these cannot be directly compared with this preclinical model.

This study supports long‐standing clinical recommendations that intact peri‐implant bone walls with a minimum thickness are essential for stability during healing. These guidelines, based on clinical observations, were formalised at the 1997–1998 ITI Consensus Conference, which recommended a minimum bone thickness of 1 mm (Buser et al. 2000). Around the same time, Spray et al. (2000) found that buccal bone walls of 1.8–2 mm reduced resorption around Brånemark‐type implants. However, bone saucerisation remained common because of the micro gap between the implant and restoration, a known site of chronic inflammation (Broggini et al. 2006; Hermann et al. 2001; King et al. 2002; Pessoa et al. 2017). In contrast, the present study excluded this variable, as implants were submerged and unrestored during healing, eliminating the influence of the micro gap.

In cases with a thin buccal bone wall, clinicians may consider strategies such as ridge reduction to increase crest width, the use of implants with a small diameter (Roccuzzo et al. 2021, 2024; Roccuzzo, Imber, Lempert, et al. 2022) or simultaneous bone augmentation procedures, as previously proposed in the literature (Buser et al. 2008; Jensen et al. 2023).

Despite its strengths and novel insights, this study has limitations. The 8‐week observation period does not capture long‐term tissue remodelling. Moreover, the sample size was based on vertical bone resorption data from a previous canine study (Monje et al. 2019), which, despite species and anatomical differences, provided the most relevant estimates. This study used miniature pigs with implants in the anterior maxilla to enable standardised surgical access, consistent implant positioning and controlled buccal bone wall thickness. The region was selected for its similarity to the human maxilla and ability to accommodate multiple implants per hemi‐maxilla. While the pig model closely mimics human bone architecture and healing, species‐specific differences must be considered when translating these findings to clinical practice (Mardas, Calciolari, and Dereka 2023; Mardas et al. 2014). Minor histometric complications occurred because of anatomical complexity near the nasal floor. All groups underwent standardised occlusal ridge flattening to achieve the target wall thickness. While this introduced minor trauma, uniform application minimised bias. The consistent association between bone resorption and initial wall thickness confirms that the procedure did not confound outcomes.

In conclusion, the present results provide preclinical evidence that implants placed in healed sites with a thin buccal bone wall (< 1 mm) are prone to buccal bone loss, potentially leading to exposure of the rough implant surface. In contrast, sites with a thick buccal bone wall (> 1.5 mm) rarely exhibit vertical bone resorption, thereby preserving the coverage of the implant surface and protecting it from the oral environment. The detailed analysis of bone remodelling and healing processes at multiple time points up to 8 weeks offers novel insights into the biological mechanisms driving these dimensional alterations and supports evidence‐based clinical decision making in implant therapy.

## Author Contributions

J.‐C.I., D.B., A.M. and A.S. conceived the ideas. J.‐C.I. and B.P. performed the clinical procedures. J.‐C.I., A.R. and D.D.B. collected the data. J.‐C.I. and D.B. led the writing. All authors contributed to interpreting the data, and read and approved the final version of the manuscript.

## Ethics Statement

The study protocol was approved by the local ethics committee of the Ministry of Food, Agriculture and Fisheries, Copenhagen, Denmark (approval number 2021‐15‐0201‐00876). It respected the Danish Animal Protection Law, adhered to the ARRIVE Guidelines and was designed and performed under consideration of the 3R (Replace, Reduce, Refine) guidelines for animal experimentation.

## Conflicts of Interest

All authors received grants from ITI (grant 1675‐2022) during the conduct of the study. D.B. has received personal fees from Straumann AG and ITI outside the submitted work. The authors declare no conflicts of interest.

## Supporting information


**Figure S1:** Representative histological section with histological landmarks. fBIC, first bone‐to‐implant contact; IS, implant shoulder; OCB, outer contour of the buccal bone wall; S, outer implant surface; SAC, start of the apical implant curvature; TP, transition point between the machined and moderately rough implant surfaces.

**Figure S2:** Representative histological sections of Group 1 (G1), Group 2 (G2) and Group 3 (G3) at time point T0 (0 days).

**Figure S3:** Representative histological sections of Group 1 (G1), Group 2 (G2) and Group 3 (G3) at time point T1 (2 weeks).

**Figure S4:** Representative histological sections of Group 1 (G1), Group 2 (G2) and Group 3 (G3) at time point T2 (4 weeks).

**Figure S5:** Representative histological sections of Group 1 (G1), Group 2 (G2) and Group 3 (G3) at time point T3 (8 weeks).

**Table S1:** Bone‐to‐implant contact evaluation.

## Data Availability

The data that support the findings of this study are available from the corresponding author upon reasonable request.
