# Supplementary figures and images for: Buccal Bone Wall Thickness Dictates the Extent of Vertical Buccal Bone Loss Following Implant Placement: A Preclinical Study
Source: J Clin Periodontol. 2025 Aug 29;52(12):1791–801. doi: 10.1111/jcpe.70027 (PMC12605777; doi:10.1111/jcpe.70027)

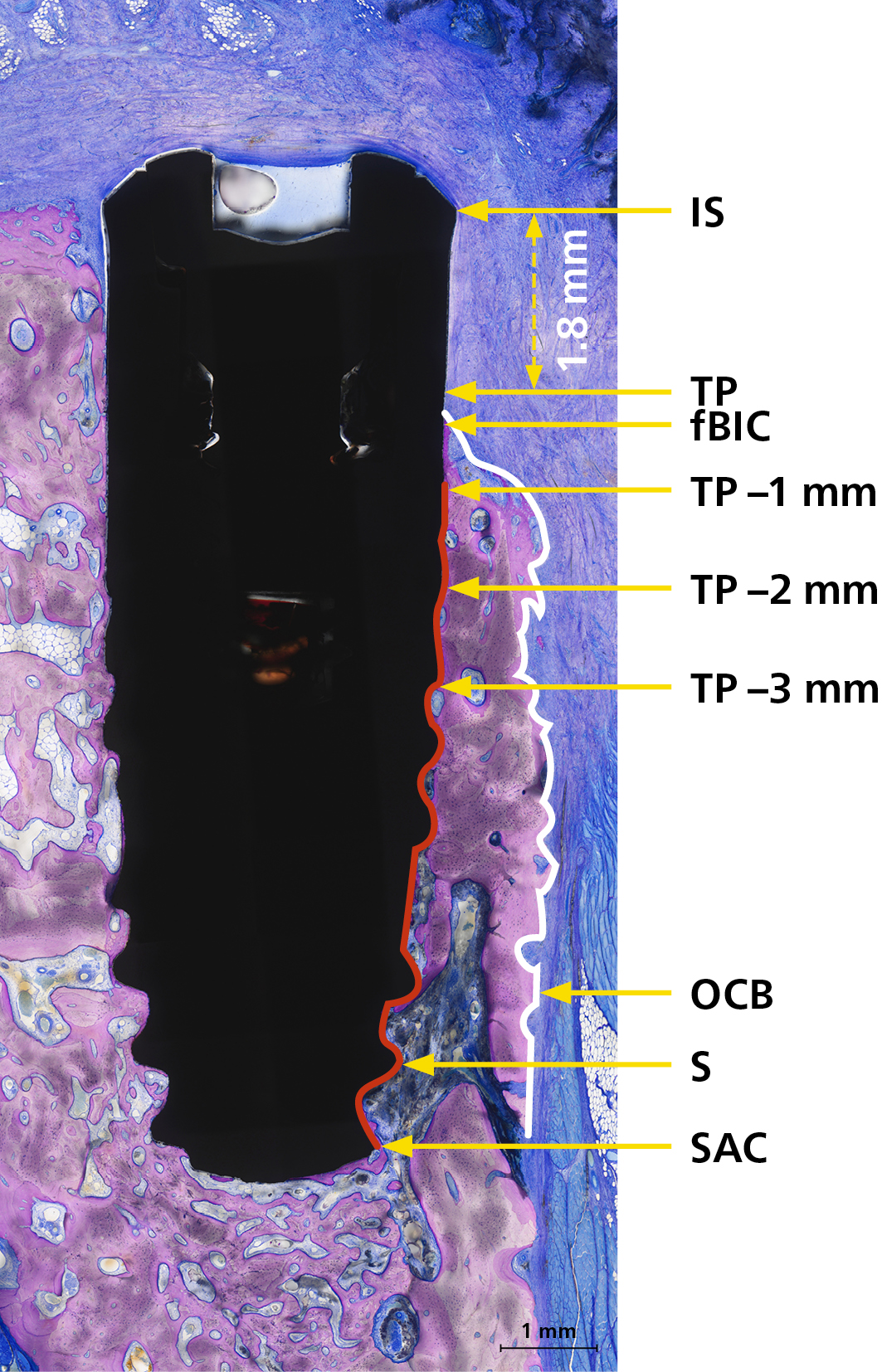

Supplement: Supplementary file 1 — Figure S1: Representative histological section with histological landmarks. fBIC, first bone‐to‐implant contact; IS, implant shoulder; OCB, outer contour of the buccal bone wall; S, outer implant surface; SAC, start of the apical implant curvature; TP, transition point between the machined and moderately rough implant surfaces. [file JCPE-52-1791-s001.jpg]

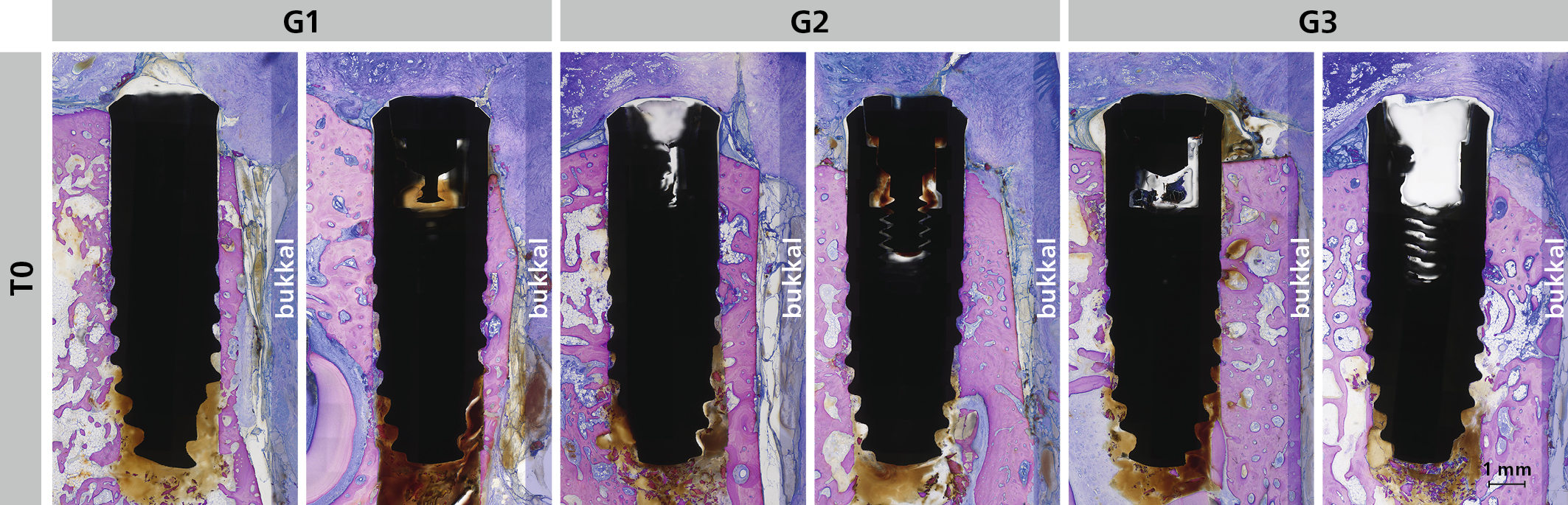

Supplement: Supplementary file 2 — Figure S2: Representative histological sections of Group 1 (G1), Group 2 (G2) and Group 3 (G3) at time point T0 (0 days). [file JCPE-52-1791-s006.jpg]

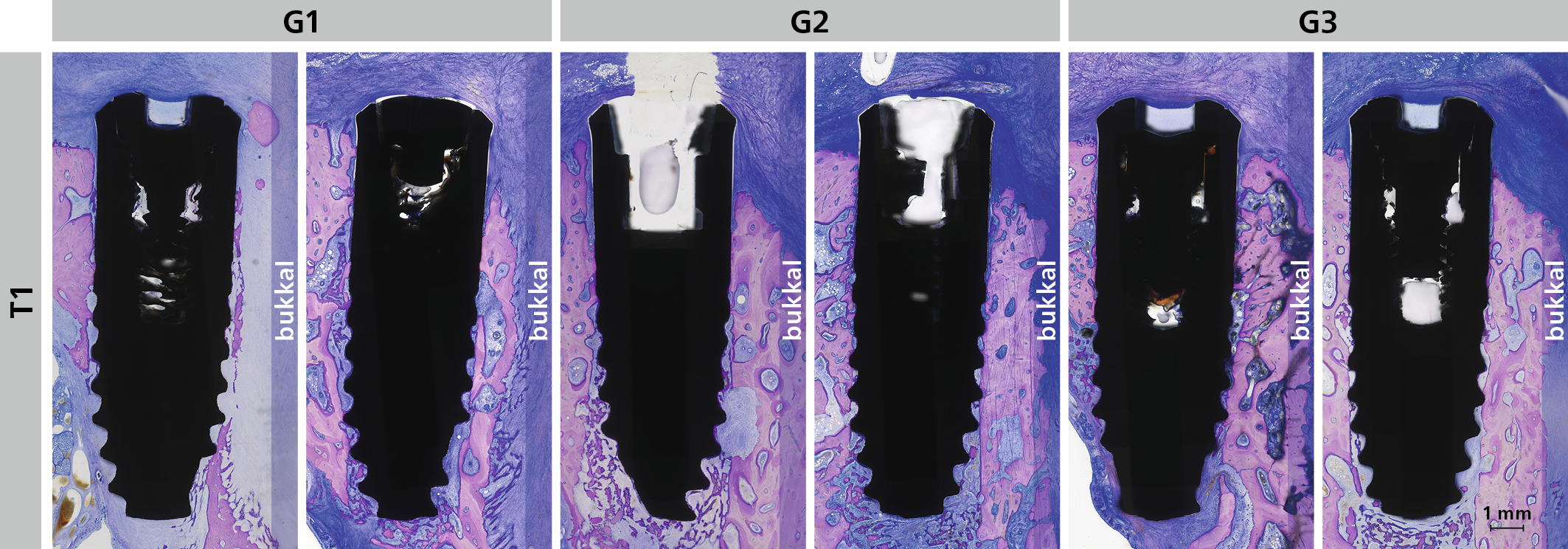

Supplement: Supplementary file 3 — Figure S3: Representative histological sections of Group 1 (G1), Group 2 (G2) and Group 3 (G3) at time point T1 (2 weeks). [file JCPE-52-1791-s004.jpg]

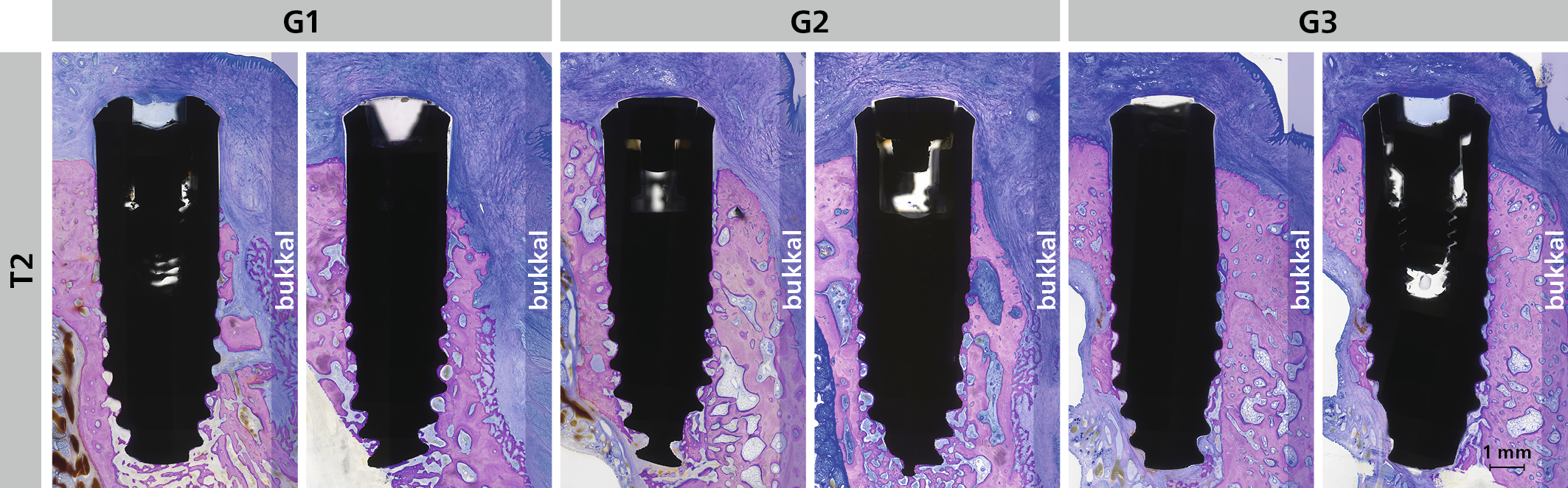

Supplement: Supplementary file 4 — Figure S4: Representative histological sections of Group 1 (G1), Group 2 (G2) and Group 3 (G3) at time point T2 (4 weeks). [file JCPE-52-1791-s005.jpg]

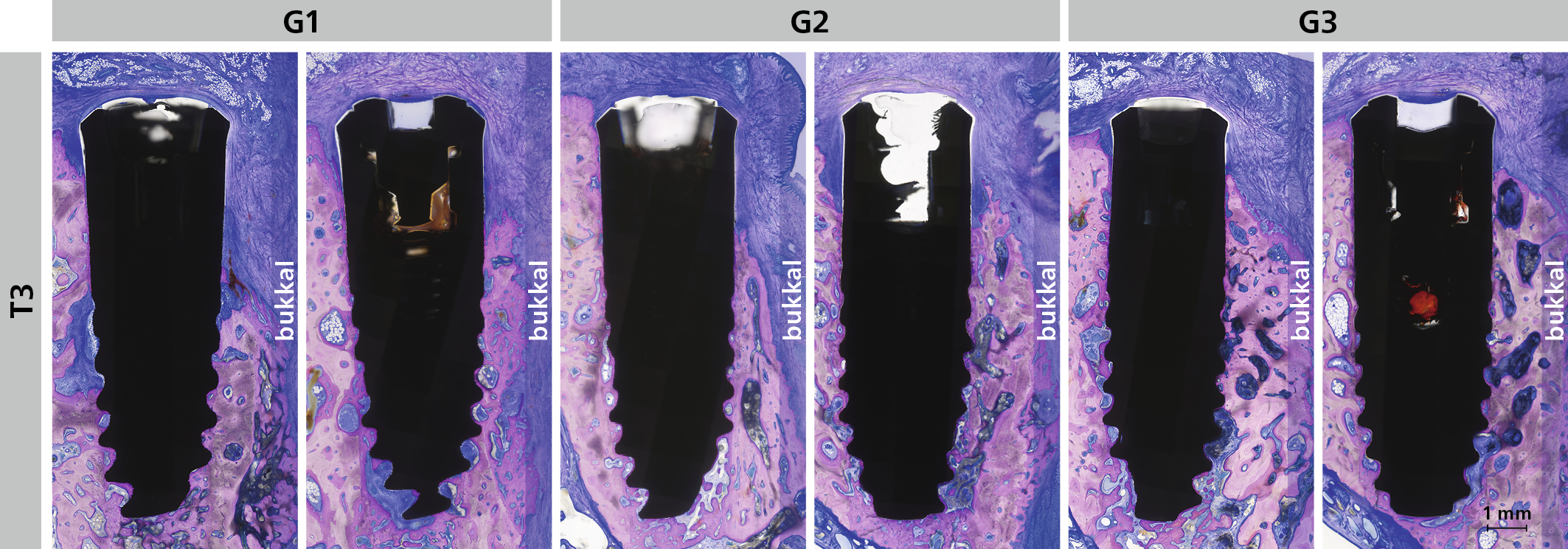

Supplement: Supplementary file 5 — Figure S5: Representative histological sections of Group 1 (G1), Group 2 (G2) and Group 3 (G3) at time point T3 (8 weeks). [file JCPE-52-1791-s002.jpg]
