# Supplementary material for: Buccal Bone Wall Thickness Dictates the Extent of Vertical Buccal Bone Loss Following Implant Placement: A Preclinical Study
Source: J Clin Periodontol. 2025 Aug 29;52(12):1791–801. doi: 10.1111/jcpe.70027 (PMC12605777; doi:10.1111/jcpe.70027)
Supplement: Supplementary file 6 — Table S1: Bone‐to‐implant contact evaluation. [file JCPE-52-1791-s003.docx]

Table S1: Bone-to-implant contact evaluation

| Group | Time | Side | Osteoid (%) | New Bone  (%) | Old Bone  (%) | Soft Tissue  (%) | Quagulum  (%) | Bone Debris  (%) | ST above fBIC  (%) | Total BIC  (%) |
| --- | --- | --- | --- | --- | --- | --- | --- | --- | --- | --- |
| 1 | 24h | total | 0 | 0 | 17.8 | 0 | 50.9 | 28.7 | 2.6 | 17.8 |
| 1 | 24h | buccal | 0 | 0 | 17.4 | 0 | 53 | 27.5 | 2.2 | 17.4 |
| 1 | 24h | palatal | 0 | 0 | 18.2 | 0 | 49 | 29.9 | 3 | 18.2 |
| 1 | 2w | total | 10.1 | 49.8 | 13.4 | 25.3 | 0 | 0 | 1.4 | 73.2 |
| 1 | 2w | buccal | 10.2 | 42.7 | 16.7 | 29.1 | 0 | 0.1 | 1.1 | 69.7 |
| 1 | 2w | palatal | 9.9 | 57.6 | 9.6 | 21.1 | 0 | 0 | 1.8 | 77.1 |
| 1 | 4w | total | 8.3 | 56.9 | 16.5 | 15.4 | 0 | 0 | 2.8 | 81.7 |
| 1 | 4w | buccal | 10.1 | 54.1 | 15.5 | 15.4 | 0 | 0 | 4.9 | 79.7 |
| 1 | 4w | palatal | 6.4 | 59.9 | 17.6 | 15.5 | 0 | 0 | 0.7 | 83.8 |
| 1 | 8w | total | 6.4 | 63.4 | 10.3 | 14 | 0 | 0 | 5.9 | 80.2 |
| 1 | 8w | buccal | 7.2 | 57.2 | 12.4 | 14 | 0 | 0 | 9.2 | 76.8 |
| 1 | 8w | palatal | 5.7 | 69.4 | 8.3 | 13.9 | 0 | 0 | 2.7 | 83.4 |
| 2 | 24h | total | 0 | 0 | 16.1 | 0 | 41.9 | 36.8 | 5.2 | 16.1 |
| 2 | 24h | buccal | 0 | 0 | 17.4 | 0 | 38.1 | 37 | 7.4 | 17.4 |
| 2 | 24h | palatal | 0 | 0 | 14.8 | 0 | 45.5 | 36.7 | 3.1 | 14.8 |
| 2 | 2w | total | 7.8 | 52.8 | 15.9 | 16.3 | 0 | 0 | 7.1 | 76.6 |
| 2 | 2w | buccal | 7 | 55.1 | 13.7 | 18.8 | 0 | 0 | 5.5 | 75.8 |
| 2 | 2w | palatal | 8.8 | 50.1 | 18.7 | 13.3 | 0 | 0.1 | 9 | 77.6 |
| 2 | 4w | total | 9.2 | 57.7 | 17.8 | 14.2 | 0 | 0 | 1.1 | 84.7 |
| 2 | 4w | buccal | 10 | 57.1 | 17.5 | 13.5 | 0 | 0 | 2 | 84.6 |
| 2 | 4w | palatal | 8.3 | 58.4 | 18.2 | 15.1 | 0 | 0 | 0 | 84.9 |
| 2 | 8w | total | 7.2 | 66.6 | 10.1 | 13.7 | 0 | 0 | 2.4 | 83.9 |
| 2 | 8w | buccal | 7.6 | 66.5 | 8.9 | 13 | 0 | 0 | 4 | 83 |
| 2 | 8w | palatal | 6.7 | 66.6 | 11.4 | 14.5 | 0 | 0 | 0.8 | 84.7 |
| 3 | 24h | total | 0 | 0 | 20.1 | 0 | 36.4 | 32 | 11.5 | 20.1 |
| 3 | 24h | buccal | 0 | 0 | 14.7 | 0 | 40.7 | 30.7 | 13.9 | 14.7 |
| 3 | 24h | palatal | 0 | 0 | 25.8 | 0 | 31.7 | 33.4 | 9.1 | 25.8 |
| 3 | 2w | total | 11.3 | 47.8 | 19.4 | 18.2 | 0 | 0.9 | 2.4 | 78.5 |
| 3 | 2w | buccal | 11.7 | 46.7 | 18.6 | 19.5 | 0 | 0.4 | 3.1 | 77 |
| 3 | 2w | palatal | 10.8 | 49.2 | 20.2 | 16.7 | 0 | 1.6 | 1.6 | 80.2 |
| 3 | 4w | total | 10.7 | 49.3 | 21.2 | 18.8 | 0 | 0 | 0 | 81.2 |
| 3 | 4w | buccal | 12.4 | 48.1 | 18.8 | 20.6 | 0 | 0 | 0 | 79.4 |
| 3 | 4w | palatal | 8.5 | 50.9 | 24.1 | 16.6 | 0 | 0 | 0 | 83.4 |
| 3 | 8w | total | 6.6 | 69.2 | 12.2 | 11.5 | 0 | 0 | 0.5 | 88 |
| 3 | 8w | buccal | 6.5 | 66.6 | 13.3 | 12.6 | 0 | 0 | 1.0 | 86.4 |
| 3 | 8w | palatal | 6.7 | 72 | 10.9 | 10.4 | 0 | 0 | 0 | 89.6 |

BIC, Bone-to-Implant Contact; fBIC, first Bone-to-Implant Contact; ST, Soft Tissue; Total BIC, osteoid + new bone + old bone.
